# Green operating room project in a multidisciplinary Surgical Unit

**DOI:** 10.1007/s13304-025-02332-9

**Published:** 2025-07-16

**Authors:** Giulia Osella, Nicola Leone, Mariachiara Benedetto, Eugenia Lavorini, Luca Petruzzelli, Alberto Arezzo, Mario Morino

**Affiliations:** 1 Department of Surgery, Annunziata hospital, Savigliano, Cuneo Italy; 2 Department of Surgical Sciences, University of Torino, C.so Dogliotti 14, 10126 Turin, Italy; 3 Department of Surgery, Azienda ospedaliera-universitaria Careggi, Florence, Italy

**Keywords:** Sustainability, Green, Sustainable surgery, Green project, Medical waste

## Abstract

The climate emergency requires effective measures to reduce the environmental impact of the healthcare system. Approximately 20% of medical waste originates from operating rooms. Proper waste segregation, along with adherence to a correct definition of biohazardous waste, are good practice procedures in the operating unit that ensure waste reduction. This study aims to assess the effectiveness of waste segregation in a multispecialty Surgical Unit. During a 3-week observational period, compared with a subsequent 3-week experimental period, counts of paper and plastic bags and the number and weight of biohazardous waste were recorded. The experimental period incorporated heightened waste sorting attention and introduced new criteria for surgical uniform disposal. While no significant differences in paper and plastic bag production were observed between the two periods, there was a non-statistically significant reduction in numbers and weight of biohazardous waste (*p* = 0.22; *p* = 0.16 respectively). Multiple regression analysis revealed a statistically significant 20 kg reduction in biohazardous waste over 3 weeks, with the same number of surgical procedures performed (*p*<0.05), resulting in 3.7 kg of biohazardous waste per surgical procedure. This reduction increased to 24 kg in the second period under the same number and type of interventions (*p*<0.05). Notably, General Surgery, Urology, Otolaryngology, and Orthopedics were identified as the surgical branches with the highest biohazardous waste production. In particular, orthopedic procedures generated 9.35 kg of hazardous medical waste per intervention, with statistical significance (*p* = 0.006). A careful separate collection of waste in the operating room, focusing on limiting biohazardous waste production, could be an important tool for reducing environmental impact and fostering economic savings. A good practice involves finding tailored solutions through teamwork as demonstrated by the present study.

## Introduction

Climate change represents one of the main threats to public health in the twenty-first century [1]. The healthcare sector is one of the main contributors to greenhouse gas emissions (over 4% of total emissions in the most advanced countries) [2].

The climate emergency requires effective measures to reduce the environmental impact of the healthcare system, as expressed in the goals of COP26 in 2021 [3, 4].

One of the main challenges in creating a low-impact healthcare system is the substantial reduction of healthcare waste [5].

Approximately 20% of healthcare waste production comes from operating rooms [6]. In fact, surgery is a sector that requires expensive equipment, advanced technologies, and high sterilization costs. Moreover, the waste generated from the operating room is considered infectious and, therefore, requires high-energy processes for safe disposal. All of this results in consuming substantial amounts of energy and producing impressive quantities of waste [5, 7].

The waste produced after a single surgery is equivalent to the weekly consumption of a family of four. In fact, on average, each surgical operation generates 5 kg of waste [8].

Studies have shown that contaminated material should account for only 6% of the annual waste production, yet often 20% of the waste is considered infectious. This results in a more significant environmental impact related to operating room activity and incurs unnecessary costs for healthcare facilities [9].

In Italy, hospital waste management is regulated by DPR 254/2003, which categorizes hazardous healthcare waste as waste contaminated with blood in quantities sufficient to make it visible or with other biological fluids. This commonly involves collecting infectious waste in yellow-colored bags and specialized rigid containers bearing the biological hazard symbol [10]. Infectious healthcare waste must be disposed of by incineration exclusively by specialized companies at a cost 10–20 times higher than ordinary waste disposal [11].

According to D.LGS 116/2020, healthcare waste, including undifferentiated and recyclable waste, if “similar in nature and composition to domestic waste”, can be considered solid urban waste and therefore disposed of according to local regulation [12].

This critical alignment with European directives on healthcare waste management enables waste recycling programs at hospitals, particularly in operating rooms.

Experts attest that merely implementing waste separation in the operating room is responsible for the highest impact on waste disposal costs.

Hospitals that provide training modules for staff and visual materials to hang in the operating room show a 30% improvement in waste separation, reducing contaminated healthcare waste by 40–59% [13, 14].

Establishing a Green Team, a group of professionals trained on the subject, is crucial. This team would plan green projects suitable for the operating room and provide ongoing leadership for their implementation [15, 16].

This study aims to evaluate the effectiveness of waste separation in a multispecialty Surgical Unit, following the guidelines outlined by a national pilot project conducted by the Department of General Surgery at the University of Turin (Italy) starting in June 2021 [14].

## Materials and methods

A prospective observational study was conducted at multispecialty Surgical Unit of the SS. Annunziata hospital (Savigliano, Cuneo, Italy), which comprises five operating rooms. Six different surgical teams (General Surgery, Otolaryngology, Urology, Orthopedics, Gynecology, and Ophthalmology) performed surgeries during the study period from September 18 to October 29, 2023.

An observational period of 3 weeks from September 18 to October 8, 2023, was compared with a second experimental period of 3 weeks from 9 to October 29 2023. During both periods, the number of paper and plastic bags and the number and weight of biohazardous waste (sorted in different bins called"Sanibox") were carried out. In addition, we recorded the number and type of surgical procedures performed daily in the Surgical Unit. Laparoscopic and open surgical procedures in an emergency or elective setting were included.

**Fig. 1:**
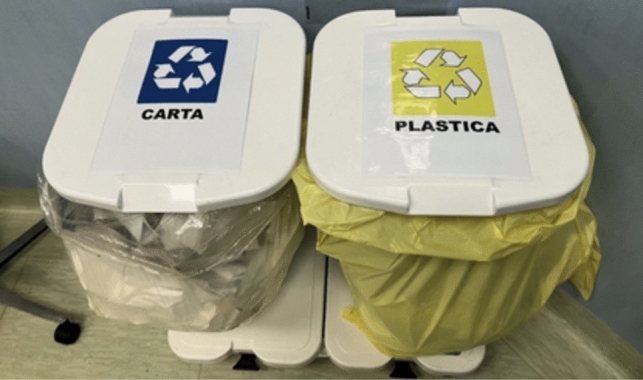
Signs posted on the paper and plastic bins located inside the operating theatre

**Fig. 2:**
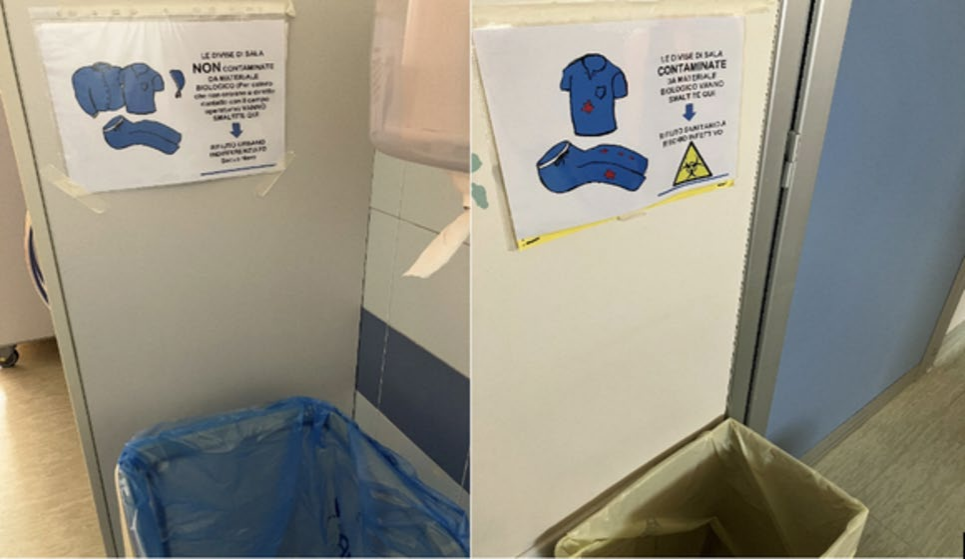
Signs for the differentiation of TNT uniforms (contaminated and non-contaminated) posted in the changing rooms of the surgical theatre

In the second period, we focused on improving the waste sorting process. Previously, the sorting was based on the color code of the recycling bag (white for paper and yellow for plastic). We placed clear signs on each bin within the Surgical Unit (Fig. 1). A new sorting criterion was introduced to reduce contaminated waste. Disposable TNT (non-woven fabric) uniforms were categorized as contaminated or non-contaminated based on whether they were worn by personnel in contact with patients. Contaminated uniforms (worn by surgeons and instrument technicians) were disposed of in Sanibox containers as biohazardous waste, while non-contaminated uniforms were disposed of as solid waste. These separate bins were placed in the changing rooms of the Surgical Unit (Fig. 2).

Establishing our “Green Team,” a dedicated group of healthcare professionals, was pivotal in this initiative. This team included the head nurse of the Surgical Unit, a professional instrument nurse, two representatives from General Surgery, two representatives from Urology, one representative each from Orthopedics, Otolaryngology, Ophthalmology, Gynecology, a delivery room nurse, a representative from Anesthesia and Intensive Care, the sterilization manager, and a hospital manager.

During Green Team meetings, key points from the literature on reducing the environmental impact associated with operating room activities were discussed according to the five pillars, or “5Rs”: Reduce, Reuse, Recycle, Rethink and Research [5]. The team also identified critical consumption areas for each surgical specialty and possible solutions.

The Green Team, tasked with promoting responsible waste management, held meetings with operating room staff to explain the necessary measures for more careful waste disposal.

The variables collected are presented as means ± standard deviation (SD), and comparisons were made using the Student’s *t* test. A linear correlation analysis was conducted between the daily weight of contaminated waste, number of surgical procedures, and period to determine the strength and direction of the relationships between these variables.

Finally, a multiple linear regression model was applied to assess the simultaneous effect of the predictors or independent variables (in this case, the period and the number or type of surgical procedures) on the quantitative variable or dependent variable (in this case, the daily weight of contaminated waste).

P values less than 0.05 were considered statistically significant. All analyses were performed using the STATA statistical package (version 14.0, Stata Corporation, College Station, Texas).

## Results

**Fig. 3:**
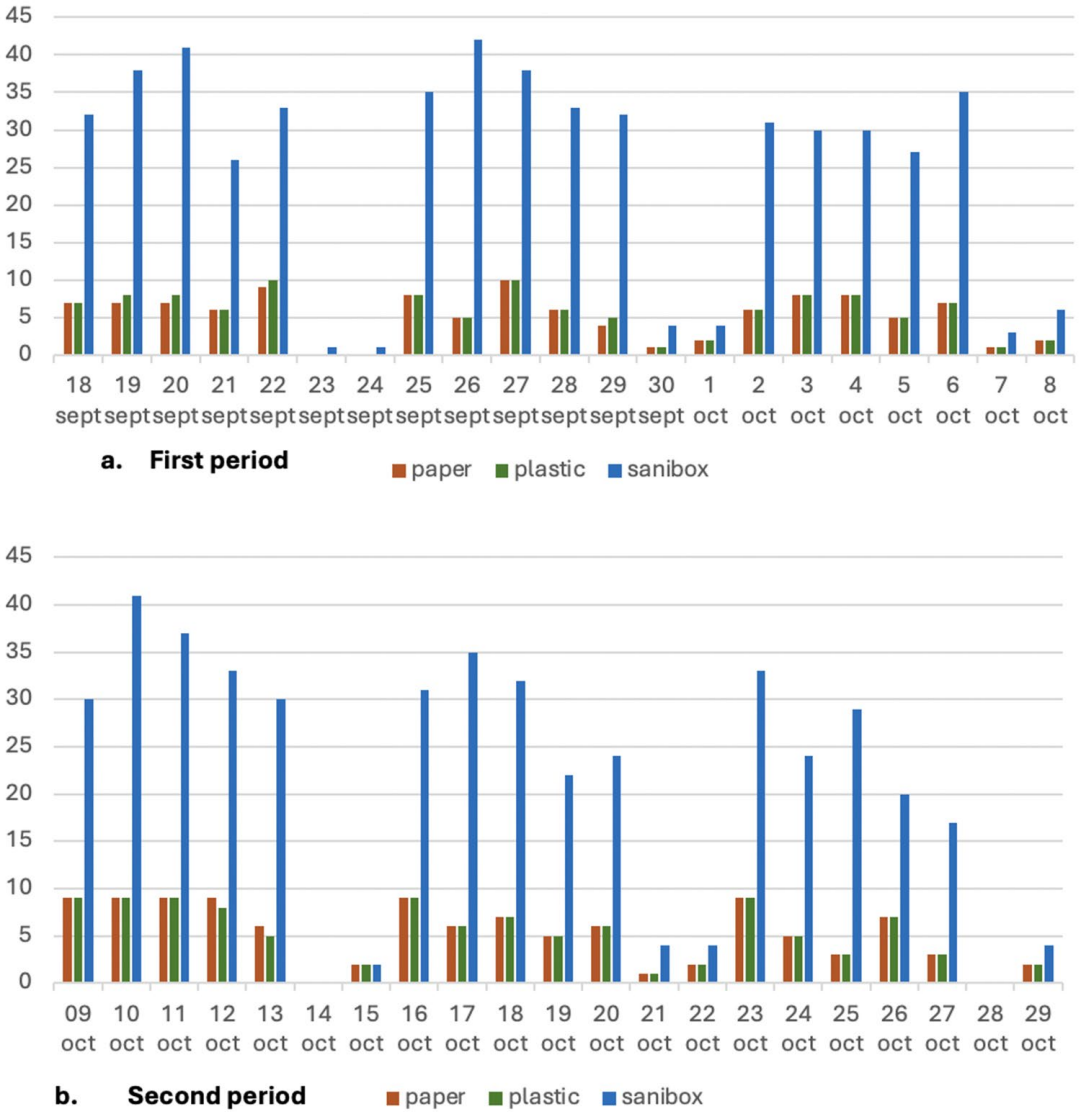
Daily distribution of the number of bags of paper, plastic, and biohazardous waste bins (Sanibox) in the first (Fig. 3a) and second (Fig. 3b) period

The distribution of the daily collection of paper and plastic bags and biohazardous waste bins during the two periods is listed in Fig. 3.

In the first period, 1834.75 kg of biohazardous waste was produced, resulting in an average of 87.37 kg daily.

The specialized company responsible for disposing of biohazardous waste charges EUR 1.12 + VAT per kg. Therefore, the average daily cost for disposal of contaminated waste during this period was EUR 97.85, with a total cost of EUR 2054.92 over 3 observational weeks. In the second period, 1522.32 kg of biohazardous waste was produced, resulting in a daily average of 72.49 kg. The average daily cost for biohazardous waste disposal during this period was EUR 81.19, with a total cost of EUR 1705 over 3 weeks.

**Fig. 4:**
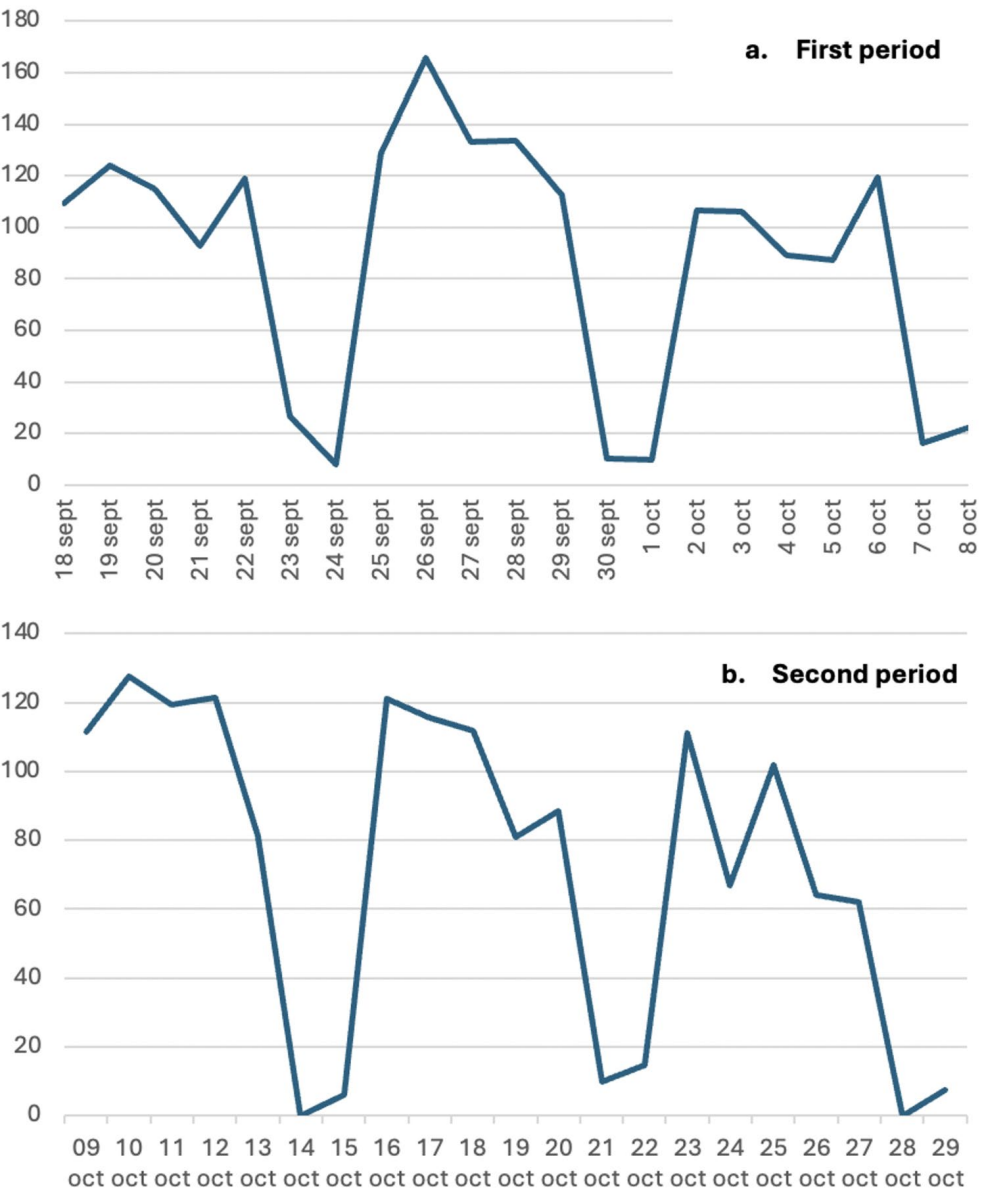
Daily distribution of the biohazardous waste weight in the first (Fig. 4a) and second (Fig. 4b) period

The daily distribution of the biohazardous waste weight in the two periods is listed in Fig. 4.

During the first period, an average of 16.52 surgical procedures were performed daily, with 347 surgical procedures in 3 weeks. During the second period, an average of 18.04 surgical procedures were performed per day, totaling 349 surgical procedures in 3 weeks.

**Fig. 5:**
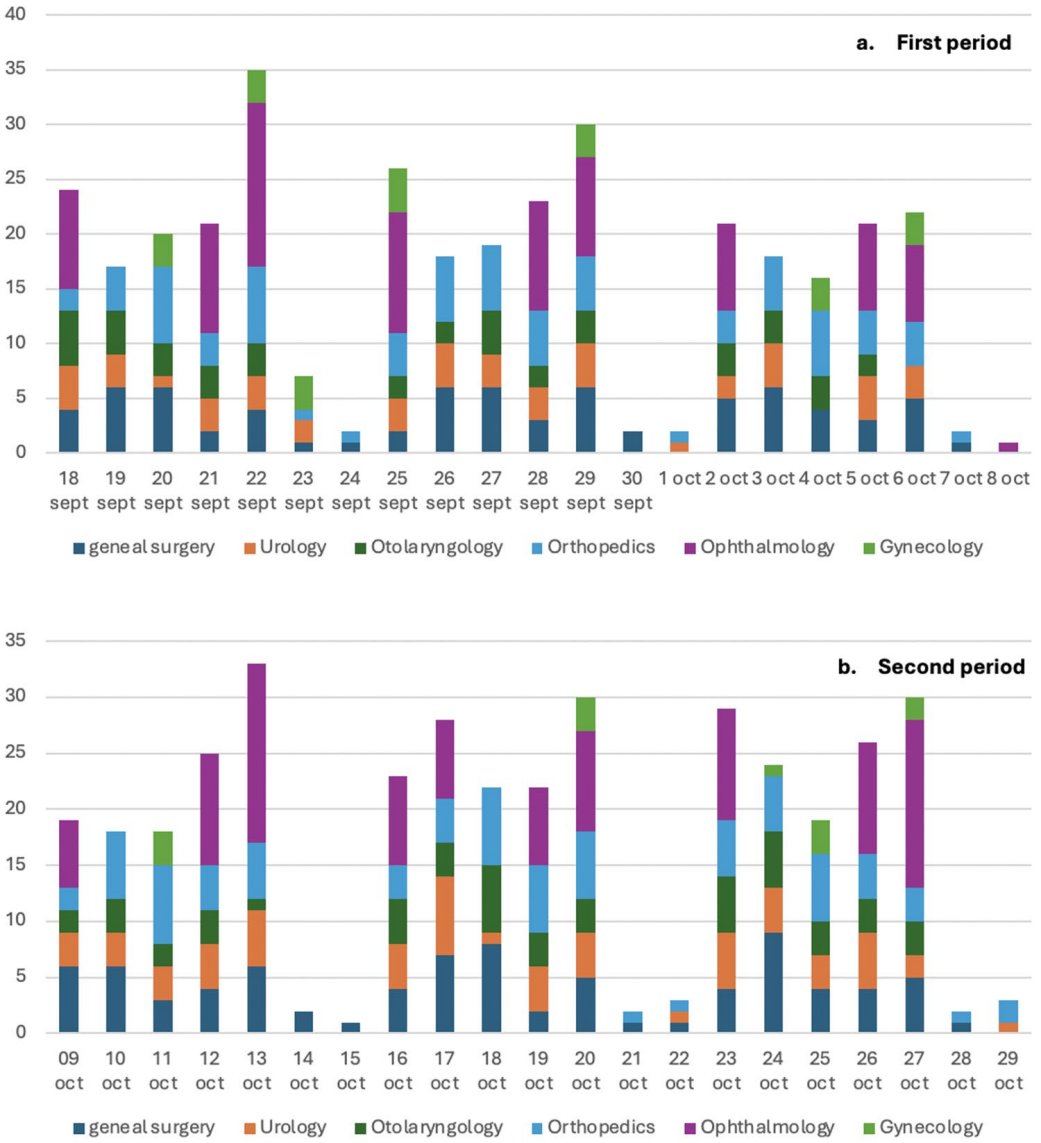
Daily number and type of surgical procedures performed during the first (Fig. 5a) and second (Fig. 5b) period

**Fig. 6:**
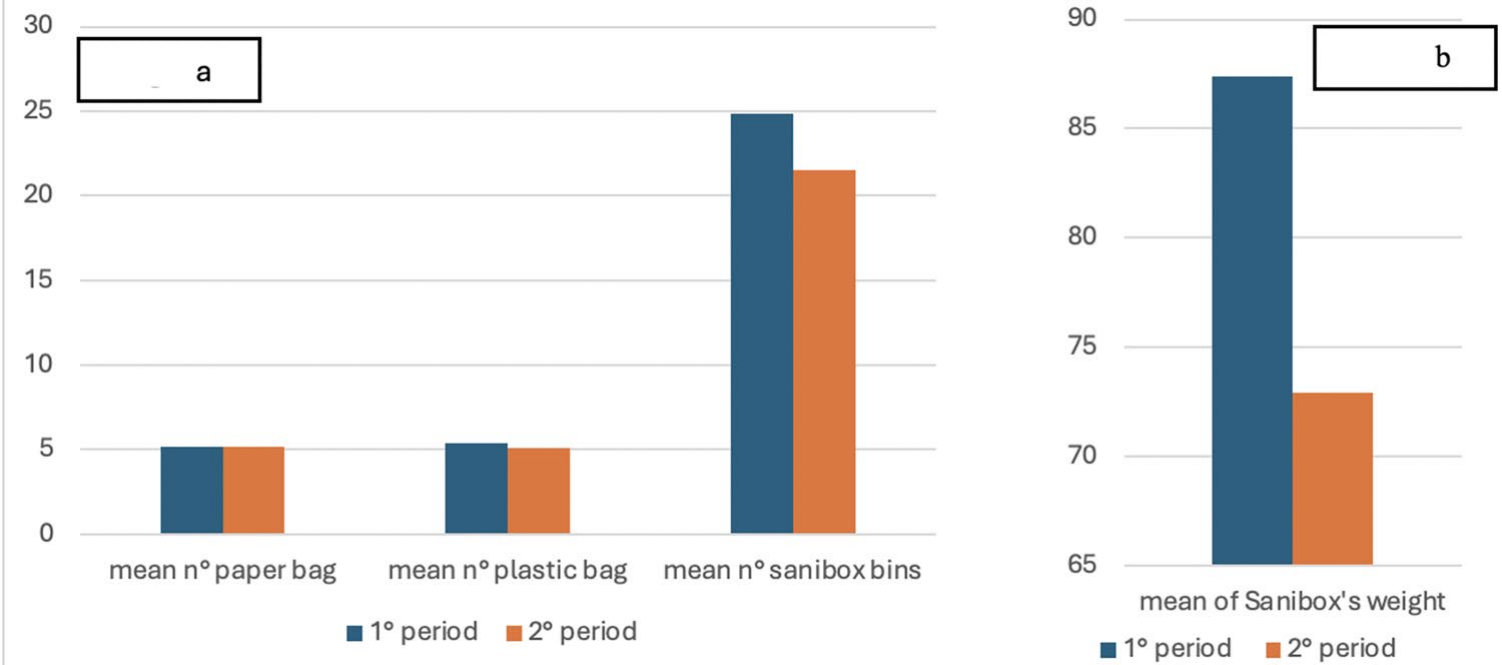
Comparison of mean per die in the two periods. Fig. 6a. Comparison of the mean number of paper bags, plastic bags and hazardous waste bins (Sanibox). Fig. 6b. Comparison of the mean of Sanibox’s weight

The daily number and type of surgical procedures performed during the two periods are listed in Fig. 5. A comparison of the two periods shows no significant difference in the number of paper bags (mean per day of 5.19 bags versus 5.19 bags) or plastic bags (mean per day of 5.38 bags versus 5.10 bags). However, biohazardous waste decreased both in terms of the number of bins (mean per day of 24.86 Sanibox bins versus 21.52 Sanibox bins) and total weight (mean per day of 87.37 Sanibox weight versus 72.49 Sanibox weight), resulting in an overall savings of 350 Euros + VAT (Fig. 6).

However, initial analysis revealed no statistically significant differences in the average values between the two study periods (*p* = 0.22 and *p* = 0.16, respectively) (Table 1).

**Table 1:** Comparison of daily means between the two periods regarding: number of paper bags, number of plastic bags, number of Sanibox bins, Sanibox’s weight, number of surgical procedures

	Period 1		Period 2			Total	
	mean	DS	mean	DS	*p*	mean	DS
N° paper bag	5,19	3,06	5,19	3,22	0,50	5,19	3,10
N° plastic bag	5,38	3,19	5,10	3,16	0,77	5,24	3,13
N° Sanibox bins	24,86	14,63	21,52	13,65	0,22	23,19	14,08
Sanibox’s weight (Kg)	87,37	49,67	72,49	47,06	0,16	79,93	48,37
N° of surgical procedures	16,52	10,00	18,04	11,07	0,68	17,29	10,44

To further assess the period’s effect on daily waste weight, we adjusted for the number of surgical procedures. A multiple regression analysis was employed to evaluate the simultaneous impact of the study period and the number of surgical procedures on daily waste weight. After accounting for the period effect, the analysis demonstrated a statistically significant correlation (*p* < 0.05) between the number of surgical procedures and biohazardous waste production. Specifically, each surgery contributed an average of 3.7 kg of biohazardous waste. Furthermore, the analysis indicated a statistically significant decrease (*p* < 0.05) of 20 kg in average daily biohazardous waste during the second period, even when performing the same number of surgical procedures (Table 2). The model’s adjusted coefficient of determination (Adj *R*^2^ = 0.648) suggests that 65% of the variability in contaminated waste weight is attributable to the different study periods and the number of surgeries performed.

**Table 2:** Linear correlation between daily weight of biohazardous waste, number of surgical procedures, and period

Biohazardous waste weight/die	COEF.	STD. ERR.	T	*P*>|T|	[95% CI]
N° of surgical procedures	3.718285	0.4303069	8.64	0.000	2.8479 4.5887
Period	−20.54262	8.881009	−2.31	0.026	−38.506 −2.5791

We also investigated which surgical branches were inherently prone to produce more contaminated waste. Our analysis showed that General Surgery, Urology, Otolaryngology, and Orthopedics were specialties prone to a higher production of biohazardous waste. Specifically, 9.35 kg of hazardous medical waste is generated, with a statistical significance (*p* = 0.006) for each Orthopedic intervention (Table 3).

**Table 3:** Multiple linear regression model to evaluate the simultaneous effect of the period, the number, and the type of surgical procedures on the daily weight of biohazardous waste

Biohazardous waste weight/die	COEF.	STD. ERR.	T	*P*>|T|	[95% CI]
General surgery	3.25234	2.929294	1.11	0.274	−2.694442 9.199122
Urology	6.303177	3.660003	1.72	0.094	−1.127023 13.73338
Otolaryngology	3.92186	4.022817	0.97	0.336	−4.244893 12.08861
Orthopedics	9.349888	3.178454	2.94	0.006	2.897283 15.80249
Ophthalmology	0.6633333	1.020211	0.65	0.520	−1.407805 2.734472
Gynecology	0.9986471	3.682667	0.27	0.788	−6.477565 8.474859

In the final model, we evaluated the effect of the considered period on the daily weight of biohazardous waste, adjusted for both the number and type of surgical procedures. The results indicate a reduction of nearly 24 kg of biohazardous waste in the second period, even when controlling for an equal number and type of procedures. This finding is statistically significant (*p* = 0.003). The adjusted R-squared coefficient of the model indicates that 80% of the variability in biohazardous waste’s weight is explained by the type of procedure and the collection period.

## Discussion

The importance of creating specific green projects for the operating room is highlighted by the fact that in our hospital, approximately 17% of the total biohazardous waste originates from the Surgical Unit’s activity. This data was obtained by comparing the production of contaminated waste generated by the entire hospital in a sample month (February 2023) with the quantity produced by the Surgical Unit in 4 weeks of study.

Our analysis revealed no statistically significant change in recycling practices within the operating room during the experimental period. While our operating room had a longstanding recycling program for paper and plastic, with specific bins already distributed, not all staff knew their presence or how to use them correctly. The bins were not adequately labeled, and there was confusion regarding the disposal of recyclable waste, as packaging often lacks clear instructions for proper recycling. By increasing attention to waste separation and introducing a simple new criterion for differentiating surgical uniforms, we achieved a statistically significant reduction in the production of contaminated waste (*p* = 0.003). This was associated with a reduction of 24 kg in contaminated waste in the second period despite performing the same number and type of surgical procedures during only 3 weeks. By extending this positive approach, we could achieve both environmental benefits and substantial economic savings.

There are numerous possible proposals, but there are just as many obstacles to overcome to reduce consumption. In a Surgical Unit, that is constantly evolving, the effort of the Green Team is fundamental to continue stimulating the search for functional methods to minimize environmental impact. The Green Team proved to be a key factor in fostering the development of new ideas for waste management and disposal [2]. During our meetings, we realized the value of interdisciplinary communication in finding collaborative strategies. Colleagues from Urology introduced a fluid management system into their operating room. This equipment collects bladder irrigation fluid during cystoscopy procedures throughout a surgical session. With a filtering mechanism, the machine empties directly into the sewer system and undergoes self-cleaning at the end of the session. Orthopedics is the branch with the highest waste production, with 9.35 kg of hazardous waste per intervention (*p* = 0.006). In response, we proposed purchasing a similar machine for use during arthroscopic procedures. This would help eliminate the significant production of biohazardous waste, such as the canisters generated during arthroscopic procedures [17, 18]. In addition, the urology group raised a significant concern that could hinder their efforts within the context of a community green project. While there is progress towards a greener operating room, the trend of new technologies increasingly favors the use of disposable instruments, especially in Urology. Some studies have shown that disposable instruments cost up to nine times more than reusable surgical equivalents, even when factoring in repair and replacement costs [18].

The General Surgery Unit proposed the establishment of a checklist in the Emergency Department to ensure that all patients empty their bladders before coming to the operating theater for urgent laparoscopic appendectomy. This simple step can help avoid on-table urethral catheterization, saving material and packaging [19]. We also hypothesized that reducing endobags for appendix and gallbladder extraction in laparoscopic procedures, when technically feasible, could further reduce waste. In addition, we instructed staff to open the laparoscopic aspirator only upon the operator’s specific request, following the ‘just-in-time’ principle [20]. Even these small measures, when implemented on a large scale, can significantly reduce waste and pollution. Certainly, a limitation of this study is its brief, 3-week duration. Despite the short duration of the study, we observed that even minor changes to waste management practices in the operating room can significantly reduce contaminated waste, leading to both environmental and economic benefits. A study with a longer observation period could certainly make these results more generalizable.

## Conclusion

This study shows that even small changes in operating room management can lead to interesting results. When staff are empowered to develop solutions for specific challenges within their environment, as they were through their participation in the Green Team, recycling programs can be an effective and cost-effective way to reduce waste in the operating room. By recognizing their role in addressing the environmental crisis, healthcare professionals can adopt ethical practices that prioritize the health and well-being of the planet.

## Data Availability

The data collected are available for reference.

## Declarations

### Human and animals research

No source of founding or research and/or publication.
